# Buccal Mucosal Grafts as a Novel Treatment for the Repair of Rectovaginal Fistulas: Protocol for an Upcoming Prospective Single-Surgeon Case Series

**DOI:** 10.2196/31003

**Published:** 2022-04-29

**Authors:** Caitlin Cahill, Natalia Kruger, John Heine

**Affiliations:** 1 Cumming School of Medicine University of Calgary Calgary, AB Canada

**Keywords:** surgical protocol, colorectal surgery, rectovaginal fistulas, fistula, surgery, gynecology, grafts, perioperative medicine

## Abstract

**Background:**

Rectovaginal fistulas (RVFs) are abnormal communications between the rectum/anus and the vagina. They are most frequently formed a result of obstetric injury and have deleterious effects on patients’ quality of life. Despite several treatment modalities, RVFs remain difficult problems to manage, and many patients fail multiple attempts at surgical repair. Buccal mucosal grafts (BMGs) may be a solution to this problem. A BMG is an oral mucosal tissue harvested from the inner cheek. There are 2 case reports that describe the successful use of BMGs in the repair of RVFs.

**Objective:**

Our objective is to validate these findings with a prospective case series while also addressing the key issues of indication, technical details, procedure safety, and short-term outcomes.

**Methods:**

A prospective single-surgeon case series will be undertaken at a university-affiliated academic tertiary care hospital in Calgary, Alberta (Canada). The estimated recruitment is between 3 and 5 patients. Patients will undergo surgical repair of their RVFs with an autologous BMG. Data on patient characteristics, fistula characteristics, and surgical variables will be collected and analyzed prospectively. The primary outcome is fistula closure. This study has been approved by the Conjoint Health Research Ethics Board at the University of Calgary (REB20-1123).

**Results:**

Two previous case reports have described the successful use of BMGs in the repair of RVFs. We have received ethics approval to attempt to validate these findings through a prospective case series.

**Conclusions:**

RVFs cause significant patient morbidity and are difficult problems to manage. Bolstered by the successful use of BMGs in urologic surgery and the previously published case reports demonstrating success in RVFs, we believe that BMGs may be a solution to RVFs.

**International Registered Report Identifier (IRRID):**

PRR1-10.2196/31003

## Introduction

### Rectovaginal Fistulas

Rectovaginal fistulas (RVFs) and anovaginal fistulas are abnormal communications between the rectum/anus and the vagina. They are most often secondary to obstetric injury, occurring in approximately 0.1% of vaginal births [1]. Other causes include infections, inflammatory and neoplastic conditions, and iatrogenic injuries. Despite their rarity, RVFs are an important problem, resulting in significant morbidity with demonstrable negative impacts on patients’ quality of life [1,2]. Despite numerous treatment modalities that have evolved over time, RVFs remain difficult problems to manage, and many patients fail several attempts at repair [1]. The procedure of choice for a simple RVF is an endorectal advancement flap, with reported success rates in the range of 41%-78% [2]. Recurrent and complex RVFs may require other surgical techniques, including fecal diversion, sphincteroplasty, muscle flaps, or even rectal resections (Multimedia Appendix 1) [3]. The variety of different techniques utilized in the surgical management of RVFs illustrates the complexity of this problem.

### Buccal Mucosal Grafts

A buccal mucosal graft (BMG) is an oral mucosal tissue harvested from the inner cheek or lower lip. It is frequently used for the repair of urethral defects, including rectourethral and vesicovaginal fistulae [4-8]. This technique was popularized after 1992 and is the first-choice graft tissue for urethroplasty in the repair of male urethral strictures [4,9] The use of oral mucosa is favored by urologists because of its similarities and compatibility with the mucosa of the urethral tract. The buccal mucosa is a nonkeratinized tissue, and its thick epithelium with vascular lamina propria gives it strength and adaptability to withstand the shearing forces in the mouth as well as defend against the microbial environment of the oral cavity [10]. Complications from BMGs are rare. A systematic review found a 4% complication rate occurring at the buccal donor site—most commonly scarring and contracture. Bleeding and hematoma formation occurred in <1% of cases. Patients can expect to have mild pain and discomfort for up to 4 weeks postoperatively. Patients may have limited range of jaw opening, but most return to preoperative range within 4 weeks [10].

### BMGs for RVFs

A systematic review of the literature was undertaken to identify whether BMGs have previously been used in the repair of RVFs. The electronic databases of Ovid MEDLINE, Embase, Cochrane Library (Cochrane Database of Systematic Reviews, Cochrane Central Register of Controlled Trials), and CINAHL (all years) were systematically searched for studies reporting on BMGs used for RVFs. The following medical subject heading terms were used: rectovaginal fistula, rectovaginal fistul* or recto-vaginal fistul* or anorectal vaginal fistul* or rectoneovaginal fistul* or recto-neovaginal fistul* or RVF or ARVF or AVF. All studies reporting on the use of BMGs for RVFs were considered eligible, and no restrictions were applied. Two case reports were identified:

1. In 2014, Grimsby et al [11] published the use of an autologous BMG in the repair of a recurrent iatrogenic RVF in a 4-year-old female. This patient’s RVF was a complication of a Soave procedure for Hirschsprung disease and failed 1 repair attempt prior to the use of the BMG.
2. In 2019, Elmer-DeWitt et al [12] described the use of an autologous BMG to repair an iatrogenic RVF in a 64-year-old transgender woman. This patient had previously undergone a penile skin inversion neovaginoplasty, which was complicated by intraoperative rectal injury.

These 2 reports demonstrated the successful use of buccal mucosa as a graft repair for an RVF. To our knowledge, these are the only such published accounts. Additional details from these case reports can be found in Table S2 of Multimedia Appendix 2.

### Idea, Development, Exploration, Assessment, Long-term Follow-up Framework for Surgical Innovation

This protocol is modelled from the IDEAL (Idea, Development, Exploration, Assessment, Long-term follow-up) framework for surgical innovation, which describes the stages of optimal surgical innovation: idea, development, exploration, assessment, and long-term follow-up [13]. The goal of this framework is to improve the quality of research in surgical and interventional procedures.

### Hypothesis and Objective

We hypothesize that an autologous BMG can successfully repair an RVF. Our objective is to validate the findings of the aforementioned case reports while also reporting on the safety, short-term outcomes, and technical details of the procedure (in keeping with the IDEAL framework for surgical innovation [13]).

## Methods

### Study Design

This study is a prospective single-surgeon case series.

### Ethics Approval

This study has been approved by the Conjoint Health Research Ethics Board at the University of Calgary (REB20-1123).

### Study Population and Recruitment

Patients will be recruited by a colorectal surgeon from a university-affiliated academic tertiary care hospital in Calgary, Alberta (Canada). Given the rarity of RVFs, the estimated recruitment is between 3 and 5 patients. The inclusion criteria are as follows: (1) female patients with a clinical or imaging diagnosis of a rectovaginal or anovaginal fistula; (2) fistula resulting from obstetrical injury, infection, inflammatory bowel disease, or radiation; (3) any number of recurrent fistulas; (4) fistula ≤2.5 cm diameter; and (5) adults ≥18 years of age. The exclusion criteria are as follows: (1) fistula resulting from neoplasia and (2) fistula >2.5 cm. Patients who meet the inclusion criteria and none of the exclusion criteria will be offered the opportunity to participate in this study. They will be provided with all the relevant information for informed consent verbally and in writing. If they decide to participate, they will be asked to sign informed consent.

### Surgical Technique

#### Donor Site Harvest

Buccal mucosa harvested from the inner cheek (vs lower lip) is recommended by the American Urological Association 2016 guidelines [4]. A local urologist experienced in BMGs will perform the buccal mucosal harvesting. Multimedia Appendix 3 describes our planned technique.

#### Fistula Repair

The technique for fistula closure/graft implantation is developed from standard techniques and practices for advancement flap closures of RVFs in addition to the work from the original 2014 case report [11]. The technique described in the 2019 case report [12] is less applicable, given the neovagina anatomy. Multimedia Appendix 4 describes our planned technique.

### Variables for Data Collection and Analysis

The variables for data collection and analysis are described in Textbox 1.

**Variables in this study.**

**Patient characteristics**

- Age
- BMI
- Number of vaginal deliveries and history of obstetrical injuries
- History of vaginal surgery
- History of anorectal surgery
- History of pelvic radiation
- Sphincter function (based on clinical examination and Cleveland Clinic Florida Fecal Incontinence Score [14])

**Fistula characteristics**

- Etiology of fistula (eg, obstetrical, infectious, inflammatory, radiation, iatrogenic)
- Location (distance from anal verge)
- Size
- Previous attempts at repair

**Surgical variables**

- Size of buccal mucosal graft
- Operative time
- Variations in surgical technique (sequential reporting, with nature and timing of modifications reported)

**Outcome variables**

- Primary outcome:
  Fistula closure at 2, 6, 12 weeks, and 1 year after the operation
- Secondary outcomes:
  Postoperative complications, including donor site morbidity (Clavien-Dindo classification, Multimedia Appendix 5).
  Postoperative sphincter function (based on clinical examination and Cleveland Clinic Florida Fecal Incontinence Score [14])

## Results

Two previous case reports have described the successful use of BMGs in the repair of RVFs. We have received ethics approval (Multimedia Appendix 6) to attempt to validate these findings through a prospective case series. This study has been approved by the University of Calgary Conjoint Research Ethics Board (REB20-1123). Plans for dissemination include publication of our results upon completion.

## Discussion

RVFs cause significant patient morbidity and are difficult problems to manage, with frequent recurrences from failed attempts at surgical repair [1]. Bolstered by the successful use of BMGs in urologic surgery and the previously published case reports demonstrating success in RVFs, we believe that BMGs may be a solution to RVFs. Historically, surgical innovation has been largely unstructured and variable, without adequate and timely evaluation [15]. This has been noted by some to have resulted in “persistent difficulties in obtaining high-quality evidence for surgical innovations” [13]. In response, recommendations for the development and assessment of new interventions have been created in the form of the IDEAL framework [15]. The 2014 and 2019 case reports describing BMG utilization in the repair of an RVF are IDEAL stage 1 (innovation) studies. Our planned case series will take on the form of an IDEAL stage 2a (development) study. As such, we plan to follow the IDEAL recommendations, which are to address the key issues of procedure safety, short-term outcomes, indications, and technical details with potential modifications.

## Appendix

Multimedia Appendix 1 Table outlining rectovaginal fistula treatment options.

Multimedia Appendix 2 Table comparing known case reports using buccal mucosal graft as treatment for rectovaginal fistulas.

Multimedia Appendix 3 Buccal mucosal graft harvest information.

Multimedia Appendix 4 Buccal mucosal graft repair of rectovaginal fistula information.

Multimedia Appendix 5 Clavien-Dindo classification system.

Multimedia Appendix 6 Ethics approval.

## Abbreviations

BMG: buccal mucosal graft
IDEAL: Idea, Development, Exploration, Assessment, Long-term follow-up
RVF: rectovaginal fistula
