# Supplementary material for: Buccal Mucosal Grafts as a Novel Treatment for the Repair of Rectovaginal Fistulas: Protocol for an Upcoming Prospective Single-Surgeon Case Series
Source: JMIR Res Protoc. 2022 Apr 29;11(4):e31003. doi: 10.2196/31003 (PMC9107045; doi:10.2196/31003)
Supplement: Multimedia Appendix 1 [file resprot_v11i4e31003_app1.docx]

Multimedia Appendix 1:

**Table S1.** Treatment options for RVF

|  | Non-operative treatment | Draining seton | Endorectal advancement flap with or without sphincteroplasty | Episioproctotomy with reconstruction | Gracilis muscle or bulbocavernous muscle (Martius) flap | Abdominal approach | Proctectomy with colon pull through or coloanal anastomosis |
| --- | --- | --- | --- | --- | --- | --- | --- |
| *Indications* | Benign or minimally symptomatic | Facilitate resolution of inflammation or infection, prevent septal abscess  Patients who are not candidates for definitive repair or with an inflammatory or neoplastic process | Procedure of choice for most simple rectovaginal fistulas | Used to repair cryptoglandular and or obstetrical fistulas. Patients with extensive sphincter defects and associated fecal incontinence | Recommended for otherwise complex rectovaginal fistulas | High rectovaginal fistulas as a result of colorectal anastomosis | Rectovaginal fistulas resulting from pelvic irradiation |
| *Grade of recommendation* | Weak recommendation based on low quality evidence | Strong recommendation based on low quality evidence | Strong recommendation based on low quality evidence | Strong recommendation based on low quality evidence | Strong recommendation based on low quality evidence | Strong recommendation based on low quality evidence | Weak recommendation based on low quality evidence |
