# Supplementary material for: Buccal Mucosal Grafts as a Novel Treatment for the Repair of Rectovaginal Fistulas: Protocol for an Upcoming Prospective Single-Surgeon Case Series
Source: JMIR Res Protoc. 2022 Apr 29;11(4):e31003. doi: 10.2196/31003 (PMC9107045; doi:10.2196/31003)
Supplement: Multimedia Appendix 2 [file resprot_v11i4e31003_app2.docx]

Multimedia Appendix 2

**Table S2.** Comparison of known case reports using BMG as treatment for RVF.

|  | Grimsby et al (2014) [11] | Elmer-DeWitt et al (2019) [12] |
| --- | --- | --- |
| **Patient Factors** | | |
| Patient Demographics | 2-year-old female | 64-year-old transgender woman |
| Past medical history/comorbidities | Hirschsprung’s disease | Prostate cancer (prostatectomy)  Hypertension  Hyperlipidemia  Remote obesity (gastric bypass surgery) |
| **Fistula Characteristics** | | |
| Etiology | Iatrogenic; complication from Suave procedure for Hirschsprung’s disease | Iatrogenic; rectal injury during neovaginoplasty |
| Fistula size | 3mm | 2-3cm |
| Fistula location | 4mm proximal to the hymenal ring | 6cm proximal to the anorectal ring |
| Previous repair attempts | One attempt, 5 months prior to BMG repair: layered closure of fistula defect with Alloderm interposition and diverting colostomy. | None |
| **BMG Repair** | | |
| Surgical position | Lithotomy | Lithotomy |
| Surgical technique | - Vaginal flap raised - Fistula resected - Mucosa closed in layers (4-0 monocryl) - Vaginal defect covered with autologous buccal mucosa graft from the lower lip (6-0 chromic) - Vaseline gauze vaginal pack | - Episiotomy incision made at the midline posterior neovagina to open the introitus - Space between neovagina and rectum infiltrated with lidocaine/epinephrine, and skin-grafted epithelium excised all the way up to the fistula - Tissue freed up 2cm cephalad to the fistula - Fistula closed in layers. - BMG harvested from inner cheek tacked down onto neovaginal posterior wall (4-0 chromic) - Vaseline gauze vaginal packing |
| Proximal diversion | Diverting colostomy | Loop ileostomy |
| Antibiotics | Not described | Pre-operative:  2g Ceftriaxone and 500mg Metronidazole  Postoperative: ceftriaxone/metronidazole continued while in hospital; prescribed trimethoprim-sulfamethoxazole on discharge (unknown duration) |
| Postoperative management | Resumed regular diet 1 day after surgery  Foley catheter and vaginal packing removed POD7  EUA 2 months after surgery with methylene blue per rectum  Colostomy reversed >2 months postoperatively | Regular diet  Foley catheter removed on POD5  Fluoroscopic images 3 months after surgery to confirm fistula closure, followed by examination under anesthesia and proctoscopy to confirm repair  Loop ileostomy reversed 3 months after surgery |
| Length of stay | 8 days | 4 days |
| Results | 100% graft take with no complications  No recurrence at 1-year postoperative | 100% graft take with no complications  No recurrence at 6 months postoperative |
