# Supplementary material for: Buccal Mucosal Grafts as a Novel Treatment for the Repair of Rectovaginal Fistulas: Protocol for an Upcoming Prospective Single-Surgeon Case Series
Source: JMIR Res Protoc. 2022 Apr 29;11(4):e31003. doi: 10.2196/31003 (PMC9107045; doi:10.2196/31003)
Supplement: Multimedia Appendix 3 [file resprot_v11i4e31003_app3.docx]

Multimedia Appendix 3: Buccal Mucosa Graft (BMG) Harvest

**Pre-operative:**

- IV antibiotics within 60 minutes of cut-time: Cefazolin (2g IV) and Metronidazole (500mg IV)

**Intra-operative:**

*Equipment:*

- 2 small right-angled retractors (i.e. Langenbeck)
- Tongue depressor (wooden; from anesthesia)
- Colorado needle tip electrocautery (set at 20/20)
- 7” toothed Gilles tissue forcep
- 20cc bottle 1% xylocaine with epinephrine; place in small cup
- 1 bottle of Bacitracin mixed with 1L of NS
- 3 or 4 small sponges
- 2 green towels
- Eye protection for patient

*Positioning:*

- Supine, head tilted to one side with endotracheal tube on opposite side

*Procedure:*

- Right-angled retractors (x2) used to retract cheek and lower lip; wooden tongue depressor used to retract tongue away from harvest site.
- Identify Stenson’s duct opposite the upper 2^nd^ molar – to be avoided during dissection
- Map out the planned graft using electrocautery, keeping 1cm away from gum line/lip/Stenson’s duct
- Raise the flap from lateral 🡪 medial, finding the plane between the mucosa/fat and the underlying muscle (masseter)
  - *There will be at least one small feeding vessel – cauterize this*
  - *There will be salivary gland ducts inferiorly – these can be divided with cautery*
- Once the graft is completely raised, place in the NS + Bacitracin solution while keeping it sterile – move to back table for fistula part of operation
- Check hemostasis
- Apply xylocaine/epi-soaked gauze on area of dissection (leave until end of case and check site)
- Defat the graft using the belly of iris scissors
- Keep graft in NS+Bacitracin solution until ready to use

**Post-operative:**

- Graft harvest site heals well by secondary intention
- Instruct patient to swish & spit 1 teaspoon of salt dissolved in a cup of water QID + PRN
- If pain uncontrolled, can give patient lidocaine spray for PRN use
- No restrictions in diet
