# Supplementary material for: Buccal Mucosal Grafts as a Novel Treatment for the Repair of Rectovaginal Fistulas: Protocol for an Upcoming Prospective Single-Surgeon Case Series
Source: JMIR Res Protoc. 2022 Apr 29;11(4):e31003. doi: 10.2196/31003 (PMC9107045; doi:10.2196/31003)
Supplement: Multimedia Appendix 4 [file resprot_v11i4e31003_app4.docx]

Multimedia Appendix 4: Buccal Mucosa Graft (BMG) Repair of Rectovaginal Fistula (RVF)

**Pre-operative:**

- Fleet enema night before surgery
- Fleet enema morning of surgery
- IV antibiotics within 60 minutes of cut-time: Cefazolin (2g IV) and Metronidazole (500mg IV)

**Intra-operative:**

*Equipment:*

- Electrocautery
- Standard anorectal tray with Pratt bivalve, toothed pick-ups
- Electrocautery
- 3-0 Vicryl
- 4-0 monocryl

*Positioning:*

- Lithotomy, yellowfin stirrups

*Procedure:*

- Perineum prepped with Betadine, including intra-vaginal and intra-rectal
- Digital rectal and vaginal examination, anoscopic and vaginal examination to identify the fistula
- Pratt bivalve used to expose the fistula in the vagina
- Posterior vaginal wall is raised up as a U-shaped flap around the fistula; the flap of mucosa discarded
- Defect (fistula) debrided and closed with 3-0 Vicryl on the vaginal side
- Wash site with Bacitracin+NS solution (from BMG harvest)
- Graft trimmed to size and laid into place, covering the closed fistula, on the vaginal side
- Graft edges are sutured to the vaginal mucosa circumferentially using 4-0 monocryl
- Vaseline gauze vaginal packing for local compression and improved graft take

**Post-operative:**

- Vaseline gauze to be gently extracted on POD1
- Patient to avoid vaginal intercourse/manipulation (i.e. tampons) for 6 weeks post-operatively
