# Supplementary figures and images for: Buccal Mucosal Grafts as a Novel Treatment for the Repair of Rectovaginal Fistulas: Protocol for an Upcoming Prospective Single-Surgeon Case Series
Source: JMIR Res Protoc. 2022 Apr 29;11(4):e31003. doi: 10.2196/31003 (PMC9107045; doi:10.2196/31003)

Multimedia Appendix 6: Ethics Approval


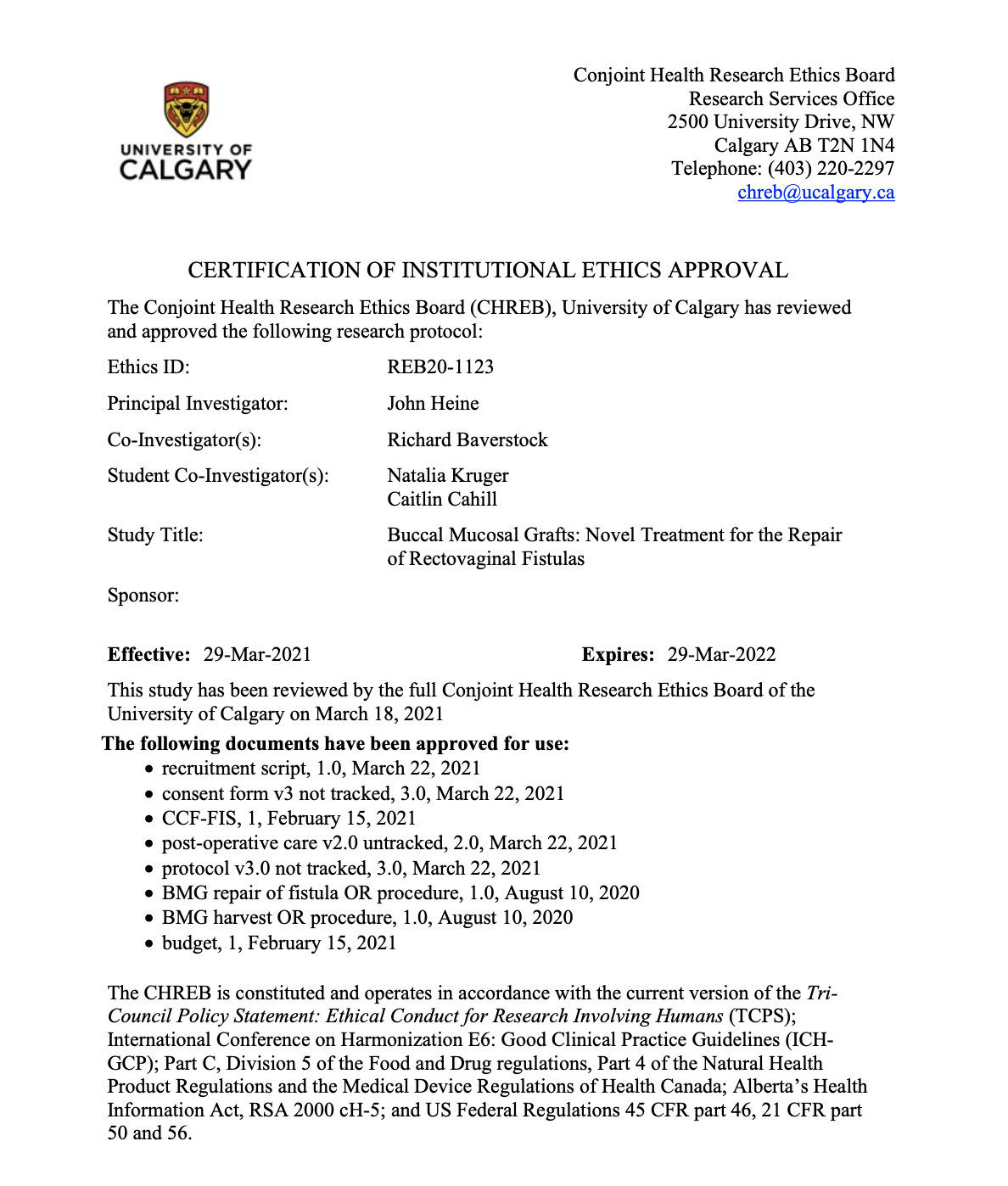


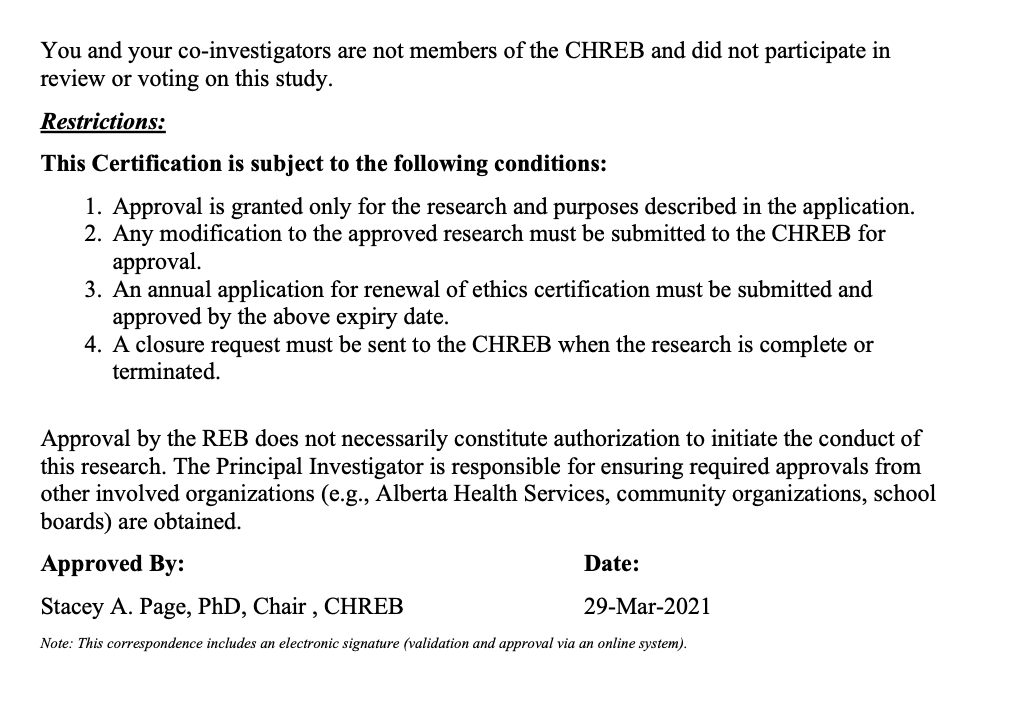

Supplement: Multimedia Appendix 6 [file resprot_v11i4e31003_app6.docx]
